# Comparative evaluation of bionote FMD NSP Ab and PrioCHECK^®^ FMDV NS Ab ELISA kits for detection of foot-and-mouth disease virus non-structural proteins antibodies in SAT serotypes

**DOI:** 10.1007/s11259-026-11536-2

**Published:** 2026-09-22

**Authors:** Khomotso Moabelo, Livio Heath, Angelika Loots, Sven Parsons, Melvyn Quan

**Affiliations:** 1 Department of Veterinary Tropical Diseases, Faculty of Veterinary Science, University of Pretoria, Pretoria, South Africa; 2 Department of Anatomy and Physiology, Faculty of Veterinary Science, University of Pretoria, Pretoria, South Africa; 3 Transboundary Animal Diseases, Onderstepoort Veterinary Institute, Agricultural Research Council, Private Bag X05, Onderstepoort, 0110 South Africa; 4 Deltamune, 34 Oxford Office Park, Bauhinia Street, Centurion, South Africa

**Keywords:** Foot-and-mouth disease, *Aphthovirus vesiculae*, diagnostic agreement, SAT serotypes

## Abstract

Foot-and-mouth disease (FMD) is a highly contagious viral disease associated with serious economic challenges. Effective serological diagnostics are essential for managing the disease properly. Detection of antibodies against non-structural proteins (NSPs) of the FMD virus (FMDV) differentiates between infected and vaccinated animals. This study evaluated the comparative performance of the commercially established PrioCHECK^®^ FMDV NS Ab ELISA, used as the reference assay, and the newer Bionote NSP Ab ELISA for the detection of FMDV NSP antibodies in cattle exposed to Southern African Territories (SAT) serotypes. A total of 401 serum samples from the Agricultural Research Council, Transboundary Animal Diseases (ARC-TAD) biobank were tested using both assays. Linear regression analysis demonstrated a significant relationship between the percentage inhibition values generated by the two assays. A moderate, positive correlation (*r* = 0.69, 95% CI: 0.61–0.74, *p* < 0.001) was observed between the two assays. Of the samples tested, 257 (64.09%) tested negative on both ELISAs, 94 (23.44%) tested positive on both, and 50 (12.46%) showed discrepant results. Among these, 38 were positive on Bionote FMD NSP Ab ELISA but negative on PrioCHECK^®^ FMDV NS Ab ELISA, while 12 were positive on PrioCHECK^®^ but negative on Bionote (χ^2^ = 12.5, *p* = 0.0004). Using the PrioCHECK^®^ FMDV NS Ab ELISA as the reference assay, the Bionote FMD NSP Ab ELISA demonstrated a sensitivity of 88.7% (95% CI: 81.1–94.0) and specificity of 87.1% (95% CI: 82.8–90.7). Receiver operating characteristic (ROC) analysis identified an optimal Bionote cut-off value of 51.825%, corresponding to a maximum Youden’s index of 0.776. Cohen’s kappa coefficient for the ELISAs was calculated to be 0.7, indicating substantial agreement between the ELISAs. The findings demonstrate that the Bionote FMD NSP Ab ELISA performs comparably to the PrioCHECK^®^ FMDV NS Ab ELISA and may serve as a useful complementary assay for FMD surveillance programmes. The availability of an additional NSP ELISA may also support testing continuity during periods of reagent shortages or supply-chain disruptions.

**Supplementary Information:**

The online version contains supplementary material available at https://doi.org/10.1007/s11259-026-11536-2.

## Introduction

Animals with cloven hooves, including cattle, pigs, sheep, goats, and several wildlife species, are susceptible to foot-and-mouth disease (FMD), a highly contagious viral disease associated with major economic losses due to reduced livestock productivity and trade restrictions (Gamaledin et al. 2019; Lv et al. 2018). A single-stranded, positive-sense RNA virus belonging to the genus *Aphthovirus* and the family *Picornaviridae*, FMD virus (FMDV) is the cause of FMD (Lv et al. 2018). In sub-Saharan Africa, the Southern African Territories (SAT) serotypes circulate predominantly in wildlife reservoirs such as African buffalo (*Syncerus caffer*), posing ongoing diagnostic and control challenges (Chitray et al. 2018; Jamal and Belsham 2018).

Serological tests that detect FMDV non-structural proteins (NSPs) antibodies are key for disease surveillance, control, and eradication programmes. These assays give vital data on infection rates, associated risk factors, and immune responses, thereby informing disease control strategies and ongoing surveillance activities. Research from different regions (Mohapatra et al. 2011; Parida et al. 2007; Sorensen et al. 2005) highlighted the importance of these tests to manage FMDV outbreaks and prevent future infections. NSP-based ELISAs are widely used in FMD surveillance programmes because they enable differentiation of infected from vaccinated animals (DIVA), as antibodies against NSPs are typically associated with viral replication rather than vaccination (Brocchi et al. 2006; WOAH 2021).

The PrioCHECK^®^ FMDV NS Ab ELISA and the Bionote FMD NSP Ab ELISA are commercially available assays designed to detect antibodies against FMDV NSPs (Bionote 2024; Thermo Fisher Scientific 2024). The PrioCHECK^®^ FMDV NSP ELISA is an established commercial assay that has been used in FMD serological surveillance and as a comparator in the validation and comparative evaluation of FMDV NSP antibody assays (Browning et al. 2020; Sharma et al. 2014; Tewari et al. 2021). The Bionote FMD NSP antibody ELISA is a more recently introduced commercial assay developed for the same diagnostic purpose (Bionote 2024).

The PrioCHECK^®^ FMDV NS Ab ELISA is currently the only NSP ELISA validated for routine use in South Africa and has been widely applied in national surveillance programmes. However, it is noteworthy that the World Organisation for Animal Health (WOAH) Terrestrial Manual on FMD explicitly advocates for the concurrent use of two NSP assays to increase confidence in serological diagnosis and surveillance outcomes. Consequently, establishing a second validated assay would be valuable (WOAH 2021). Reliance on a single commercial assay may present challenges related to assay availability, continuity of testing, and supply-chain disruptions. Consequently, the evaluation of alternative NSP ELISAs, such as the Bionote FMD NSP Ab ELISA, is important to support surveillance capacity and provide an additional testing option for FMD control programmes.

In South Africa, FMD is controlled under the Animal Diseases Act (Act 35 of 1984), and most of the country has been designated as a FMD-free zone without vaccination by the WOAH (Bruckner et al. 2002). The country’s FMD control zones are categorised into four areas: the FMD infected zone, the FMD protection zone, the FMD high surveillance area and the FMD free zone (Department of Agriculture 2025). However, an FMD outbreak in 2019 in Limpopo Province, outside the protection zone, led to the loss of FMD-free zone status as recognised by WOAH (DAFF 2019). More recently, widespread and ongoing outbreaks of FMD have been reported across all nine provinces of South Africa, predominantly associated with serotypes SAT-1 and SAT-2, with the highest burden observed in Free State, KwaZulu-Natal, Gauteng, and North West provinces. The current epidemiological situation reflects a large number of active outbreaks and limited resolution, indicating sustained transmission and continued viral circulation (Department of Agriculture 2025). Under these circumstances, the availability of multiple validated NSP ELISAs would strengthen South Africa’s serological surveillance capacity and improve resilience during outbreak investigations and post-outbreak monitoring.

This study aimed to evaluate the comparative diagnostic performance of the Bionote FMD NSP Ab and PrioCHECK^®^ FMDV NS Ab ELISA kits by assessing the level of agreement between the two assays using sera from cattle with positive, negative and inconclusive serological results. Rather than undertaking a full diagnostic validation against an independent biological reference standard, the study was designed to determine the degree of equivalence between the Bionote FMD NSP Ab assay and the PrioCHECK^®^ FMDV NS Ab assay, which is currently the only NSP ELISA validated for routine use in South Africa.

## Materials and methods

### Sample selection

The study’s sample size was estimated at 423 using an online sample size estimator for Cohen’s kappa (Donner and Eliasziw 1992; Shoukri et al. 2004). Parameters included a minimum acceptable kappa (ƙ_0_) of 0.7, and expected kappa (ƙ_1_) of 0.8, proportion of outcome (p) of 0.5, a significance level (𝛼) of 0.05, a power (1-ꞵ) of 80%, and expected dropout rate of 5%. The κ₀ value of 0.7 was selected as the minimum acceptable level of substantial agreement for comparable diagnostic performance, while κ₁ = 0.8 represented the expected level of agreement based on previous comparative evaluations of NSP ELISAs used in FMD surveillance (Viera and Garrett 2005).

Serum samples used in this study were obtained from the biobank of the Transboundary Animal Disease (TAD) section of Onderstepoort Veterinary Research (OVR), Agricultural Research Council (ARC), South Africa. The samples originated from unvaccinated cattle as part of FMD sero-surveillance, where approximately 30 samples per herd were collected. Samples were collected from farms and dip tanks in areas with reported outbreaks associated with FMDV SAT-1, SAT-2, and SAT-3 serotypes.

The TAD diagnostic laboratory uses the Solid Phase Competition ELISA (SPCE) to detect antibodies against FMDV structural proteins. Fifteen batches of sera (*n* = 469) were purposively selected from the biobank based on SPCE results: positive (0–80%; *n* = 105), inconclusive (*n* = 45), and negative (*n* = 319). The SPCE-positive results were interpreted as evidence of natural infection rather than vaccination-induced antibodies, as vaccination against FMDV was not practised outside the FMD Control Zones at the time the samples were collected.

A total of 68 samples were excluded because of contamination, moderate to severe haemolysis, or insufficient sample volume, resulting in a final study population of 401 serum samples. Testing was performed by a single operator (KM), who was blinded to both the SPCE batch classifications and the individual sample results.

### FMD NSP ELISAs

All samples and reagents were brought to room temperature before testing, and sera were briefly vortexed and centrifuged. Samples were tested in duplicate for antibodies against FMDV NSP using the PrioCHECK FMD NS Ab ELISA strip kit (Thermo Fisher Scientific, Waltham, MA, USA) and Bionote FMD NSP Ab ELISA (Bionote Inc., South Korea) according to the manufacturer’s instructions. For the PrioCHECK FMD NS Ab ELISA, serum samples were tested at a final dilution of 1:2.6 by adding 50 µL of serum to 80 µL of ELISA buffer, using the single-day serum incubation protocol. For the Bionote FMD NSP Ab ELISA, serum samples were tested at a final dilution of 1:2 by adding 50 µL of serum into the appropriate wells, followed by 50 µL of working enzyme conjugate. Appropriate kit and internal laboratory controls were included on each test plate. Optical density (OD) was measured at 450 nm using a Multiskan RC microplate reader (Labsystems, Finland).

For the PrioCHECK FMDV NS Ab ELISA, results were interpreted based on percentage inhibition (PI) calculated according to the manufacturer’s formula. Samples with PI < 50% were classified as negative, and those with PI ≥ 50% as positive. For assay validity, the maximum OD450 (mean OD450 of negative control) had to exceed 1.0, the mean PI of weak positive control had to exceed 50%, and the average PI of positive control had to exceed 70%. All plates met the acceptance criteria. For the Bionote FMD NSP Ab ELISA, results were similarly interpreted based on PI calculated according to the manufacturer’s formula. Samples were classified as negative when PI < 50%, and positive if the PI ≥ 50%. For valid test results, the mean OD of the negative control had to be ≥ 1.0, and the mean OD of the positive control had to fall within the acceptable range (-0.005–0.5000). All plates met the criteria specified in the protocol.

### Statistical analysis

The ELISA results were recorded in Microsoft Excel 2016 (version 2603, Microsoft, Redmond, WA, USA) and analysed using IBM SPSS Statistics (version 30, IBM Corp, Armonk, NY, USA) and Epitools (https://epitools.ausvet.com.au/).

The normality of the data distribution was evaluated using the Shapiro-Wilk test of normality. Linear regression and Pearson correlation analyses were used to assess the relationship between PI values generated by the Bionote FMD NSP Ab ELISA and the PrioCHECK^®^ FMDV NS Ab ELISA. Correlation coefficients were interpreted according to the guidelines proposed by Schober et al. (2018).

Using the PrioCHECK^®^ FMDV NS Ab ELISA as the reference assay, the diagnostic sensitivity, specificity, positive predictive value (PPV), and negative predictive value (NPV) of the Bionote FMD NSP Ab ELISA were calculated with corresponding 95% confidence intervals. Receiver operating characteristic (ROC) analysis was performed to evaluate the diagnostic performance of the Bionote assay and to identify the optimal cut-off value using Youden’s Index.

Agreement between the two assays was evaluated using overall percentage agreement, positive agreement, negative agreement, and Cohen’s kappa (κ) statistic with 95% confidence intervals. Kappa values were interpreted according to the classification proposed by Viera and Garrett (2005). McNemar’s Chi-squared test was used to assess differences in the proportions of discordant results between the two assays. Statistical significance was set at *p* < 0.05.

## Results

**Fig. 1:**
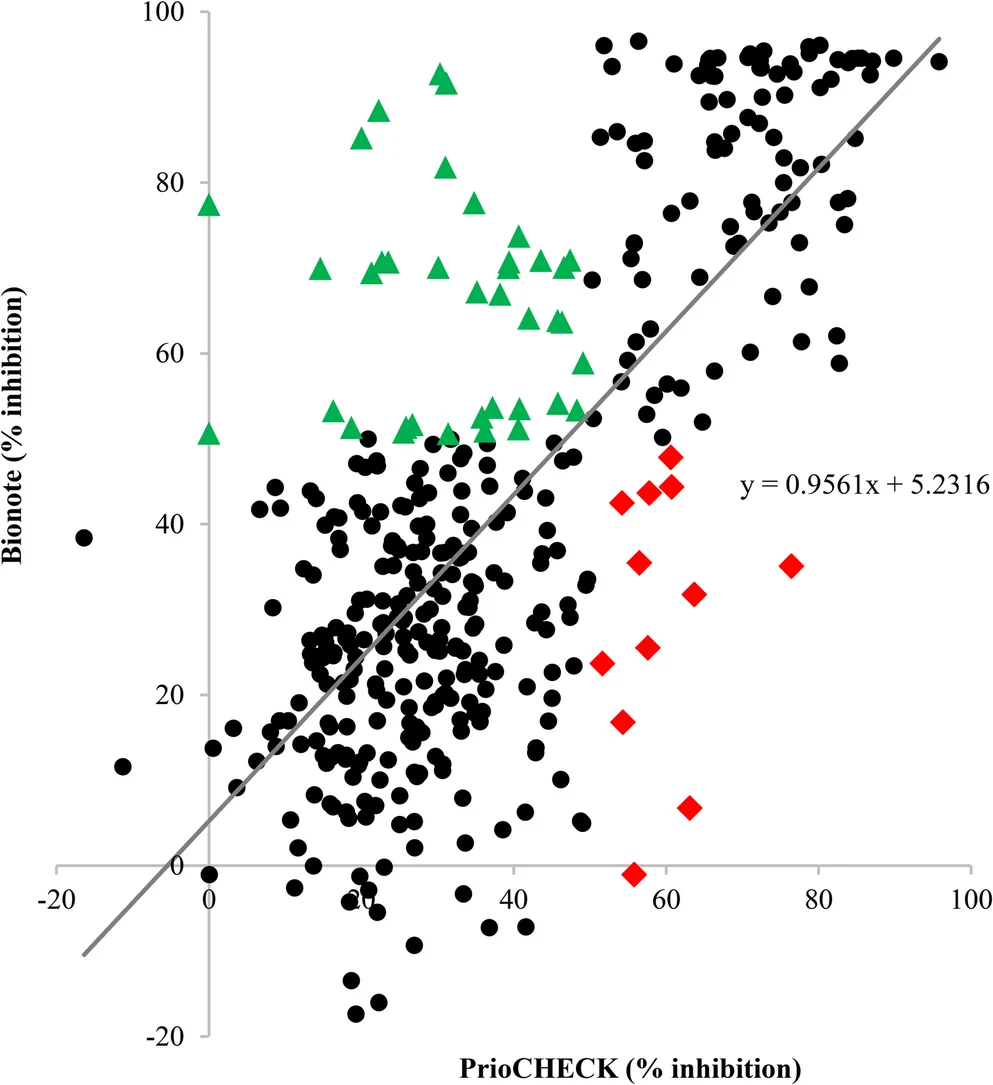
Relationship between percentage inhibition (PI) values obtained using the Bionote FMD NSP Ab ELISA and the PrioCHECK^®^ FMDV NS Ab ELISA. Black circles represent concordant results, green triangles represent Bionote-positive/PrioCHECK-negative samples, and red diamonds represent PrioCHECK-positive/Bionote-negative samples. The solid grey line represents the fitted linear regression model

Linear regression analysis demonstrated a significant relationship between the PI values obtained using the Bionote FMD NSP Ab ELISA and the PrioCHECK^®^ FMDV NS Ab ELISA (Fig. 1). The regression model was statistically significant (F = 399.552, *p* < 0.001) and explained 50.0% of the variation in PrioCHECK^®^ FMD NS Ab ELISA PI values (R² = 0.500). The fitted regression equation was: PrioCHECK FMD NS Ab ELISA PI = 16.180 + 0.523 × Bionote FMD NSP Ab ELISA PI. Pearson correlation analysis showed a statistically significant moderate positive correlation between the two assays (*r* = 0.69, 95% CI: 0.61–0.74, *p* < 0.001, *n* = 401).

A Pearson correlation test showed a statistically moderate, positive correlation between the results obtained from the assays results (*r* = 0.69, 95% CI: 0.61–0.74, *n* = 401, *p* < 0.001). Correlation coefficients were interpreted using established guidelines proposed by Schober et al. (2018), where values of 0.00-0.10, 0.10–0.39, 0.40–0.69, 0.70–0.89, and 0.90-1.00 indicate negligible, weak, moderate, strong, and very strong correlation, respectively.

To further characterise the study population, the NSP ELISA results were compared with the available SAT-2 SPCE results (Supplementary Tables S1 and S2). Among the 57 SAT-2 SPCE-positive samples, 38 (66.7%) and 40 (70.2%) were positive by the PrioCHECK^®^ FMDV NS Ab ELISA and Bionote FMD NSP Ab ELISA, respectively. Of the 26 SAT-2 SPCE-inconclusive samples, 10 (38.5%) and 9 (34.6%) were positive by the PrioCHECK^®^ and Bionote NSP ELISAs, respectively. Among the 258 SAT-2 SPCE-negative samples, 25 (9.7%) were positive by the PrioCHECK^®^ FMDV NS Ab ELISA, whereas 48 (18.6%) were positive by the Bionote FMD NSP Ab ELISA. Not all serum samples were tested against all three SAT serotypes by SPCE. Most available SPCE data were generated using the SAT-2 assay, whereas only a limited number of samples were tested using the SAT-1 and SAT-3 assays, all of which yielded negative results. Consequently, only the SAT-2 SPCE data are presented in the supplementary analyses. As NSP ELISAs detect antibodies against FMDV non-structural proteins irrespective of serotype, these comparisons are intended to provide epidemiological context for the sampled population rather than to evaluate assay performance according to serotype.

**Fig. 2:**
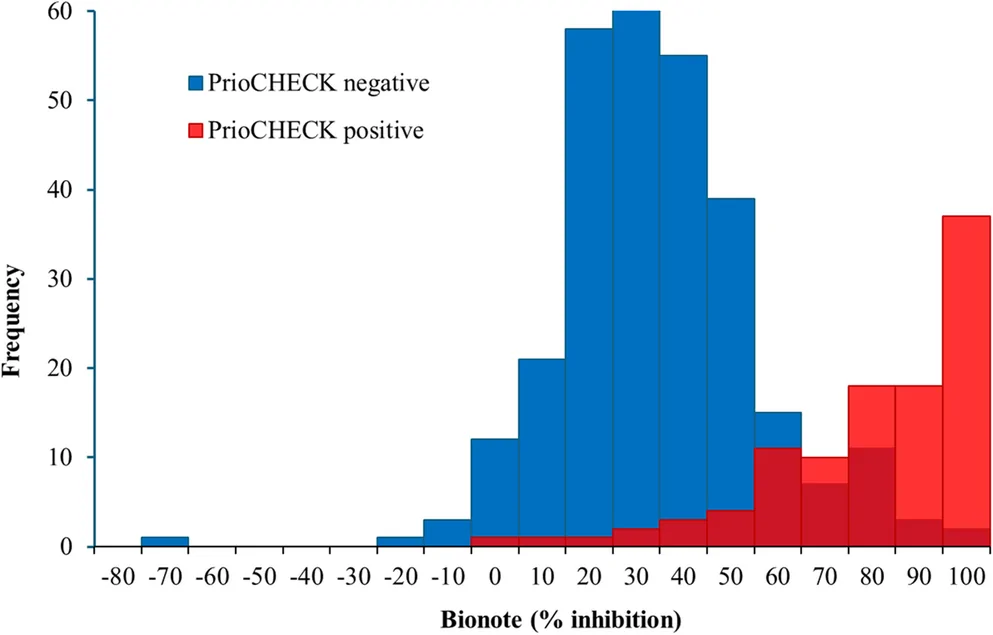
Distribution of Bionote FMD NSP Ab ELISA percentage inhibition (PI) values according to PrioCHECK^®^ FMDV NS Ab ELISA classification. Blue bars represent samples classified as negative by the PrioCHECK^®^ FMDV NS Ab ELISA, whereas red bars represent samples classified as positive. The overlap between distributions around the diagnostic threshold illustrates the potential influence of cut-off selection on assay classification

The distribution of Bionote FMD NSP Ab ELISA PI values according to PrioCHECK^®^ FMDV NS Ab ELISA classification is shown in Fig. 2. Samples classified as positive by the PrioCHECK^®^ FMDV NS Ab ELISA generally exhibited higher Bionote PI values, whereas PrioCHECK^®^-negative samples were concentrated at lower inhibition values. Although some overlap between the groups was observed around the diagnostic cut-off region, the distribution demonstrates clear separation between the majority of positive and negative samples.

**Fig. 3:**
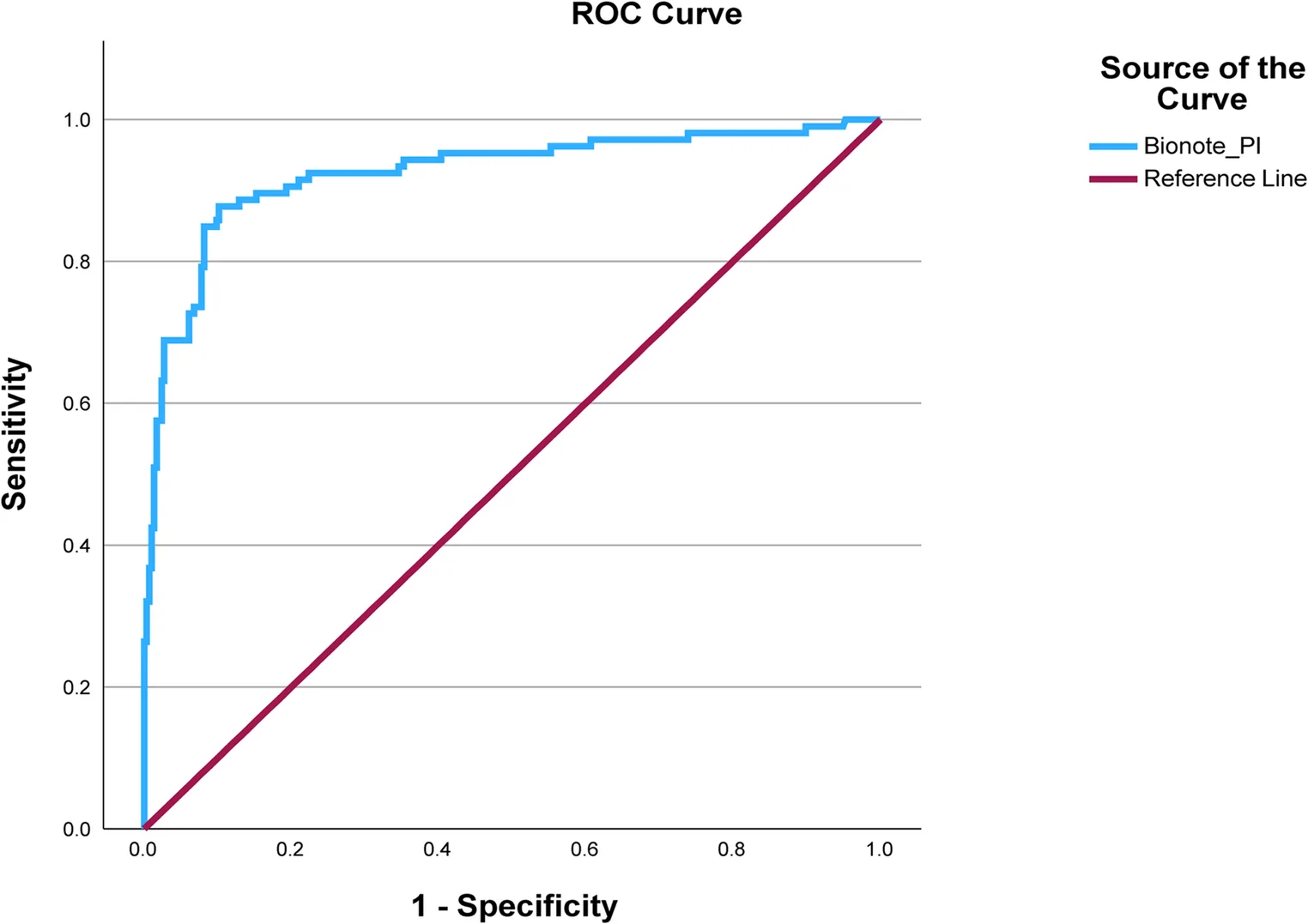
Receiver operating characteristic (ROC) curve for the Bionote FMD NSP Ab ELISA using the PrioCHECK^®^ FMDV NS Ab ELISA as the reference assay. The curve illustrates the trade-off between sensitivity and specificity across different PI cut-off values. The optimal cut-off value identified by Youden’s index was 51.83%

A ROC analysis was performed using the PrioCHECK^®^ FMDV NS Ab ELISA as the reference method assays (Fig. 3). The analysis identified an optimal Bionote cut-off value of 51.83%, corresponding to the maximum Youden’s Index of 0.776. This threshold resulted in improved sensitivity and specificity relative to the manufacturer’s recommended cut-off, suggesting that assay agreement may be influenced by threshold selection.

Using the PrioCHECK^®^ FMDV NS Ab ELISA as the reference assay, the Bionote FMD NSP Ab ELISA correctly identified 94 of 106 positive samples and 257 of 295 negative samples (Table 1). The sensitivity and specificity of the Bionote FMD NSP Ab ELISA were 88.7% (95% CI: 81.1–94.0) and 87.1% (95% CI: 82.8–90.7), respectively. The PPV and NPV were 71.2% (94/132) and 95.5% (257/269), respectively. Overall concordance between the two assays was 87.5% (351/401).

**Table 1:** Contingency table of the results obtained from testing 401 sera samples with a PrioCHECK ^®^ FMDV NS Ab ELISA and a Bionote FMD NSP Ab ELISA

		PrioCHECK^®^ FMDV NS Ab ELISA		
		Positive	Negative	Total
Bionote FMD NSP	Positive	94	38	132
Ab ELISA	Negative	12	257	269
	Total	106	295	401

The proportion of positive agreement between the assays was 0.7899, while the proportion of negative agreement was 0.9113. Cohen’s Kappa coefficient was 0.70 (95% CI: 0.63–0.78, z = 14.23, *p* < 0.001). Cohen’s kappa values were interpreted according to the classification proposed by Viera and Garrett (2005), where values of 0.00-0.02, 0.21–0.40, 0.41–0.60, 0.61–0.80, and 0.81-1.00 indicate slight, fair, moderate, substantial, and almost perfect agreement, respectively.

A McNemar’s Chi-squared test showed a significant difference in the proportions of discordant results between the two assays. (χ^2^ = 12.5, *p* = 0.0004). Of the 50 discrepant samples 12.5%, 38 (76.0%) tested positive on the Bionote FMD NSP Ab ELISA but negative on the PrioCHECK^®^ FMDV NS Ab ELISA assay, whereas 12 (24.0%) tested positive on the PrioCHECK^®^ FMDV NS Ab ELISA assay but negative on the Bionote FMD NSP Ab ELISA assay.

## Discussion

The findings should be interpreted within the context of the study objective, which was to evaluate the comparative performance of the Bionote FMD NSP Ab ELISA relative to the PrioCHECK^®^ FMDV NS Ab ELISA rather than to conduct a full diagnostic validation against an independent biological reference standard. Overall, the results demonstrated substantial agreement between the two assays when testing sera from cattle exposed to FMDV SAT serotypes under field surveillance conditions in South Africa. Together, the regression, ROC, and agreement analyses demonstrated that the Bionote FMD NSP Ab ELISA performed comparably to the PrioCHECK^®^ FMDV NS Ab ELISA assay under these conditions.

Comparison of the NSP ELISA results with the available SAT-2 SPCE data showed that most SPCE-positive samples were also positive by both NSP assays. These analyses were included to further characterise the serological profile of the study population; however, direct comparisons should be interpreted cautiously because the SPCE and NSP ELISAs detect antibodies against different viral antigens. Furthermore, the NSP assays are not serotype-specific and therefore cannot be used to assess assay performance according to individual SAT serotypes.

Linear regression and Pearson correlation analyses demonstrated a significant positive relationship between the PI values generated by the Bionote FMD NSP Ab ELISA and PrioCHECK^®^ FMDV NS Ab ELISA. The observed Pearson correlation coefficient of 0.69 indicated a moderate positive association between the two assays, suggesting that samples with higher PI values in one assay generally produced higher PI values in the other. Dill et al. (2017) reported near-perfect agreement between the PrioCHECK^®^ FMDV NS Ab ELISA and the ID Screen^®^ FMD NSP ELISA (*r* = 0.97), which was higher than the correlation observed in the present study. These differences may reflect variations in assay design, sample populations, circulating serotypes, and epidemiological conditions. Nevertheless, the substantial agreement observed in the current study indicates that the Bionote FMD NSP Ab ELISA performs comparably to the PrioCHECK^®^ FMDV NS Ab ELISA under South African field conditions.

The ROC analysis provided additional evidence of the comparative performance of the Bionote FMD NSP Ab ELISA. The high AUC value (0.925) demonstrated excellent discrimination between samples classified as positive and negative by the PrioCHECK^®^ FMDV NS Ab ELISA. Furthermore, optimisation of the Bionote FMD NSP Ab ELISA cut-off value to 51.825%, corresponding to the maximum Youden’s index of 0.776, resulted in modest improvements in both sensitivity and specificity. These findings suggest that assay performance may be influenced by threshold selection and that cut-off optimisation may be beneficial when the assay is applied under specific epidemiological or surveillance conditions. However, the manufacturer’s recommended cut-off also demonstrated good overall performance and remains appropriate for routine diagnostic use.

Using the PrioCHECK^®^ FMDV NS Ab ELISA as the reference method, the Bionote FMD NSP Ab ELISA demonstrated high relative sensitivity (88.7%) and specificity (87.1%), indicating a strong ability to correctly classify both positive and negative samples. The overall concordance between the two assays was 87.5%, further supporting their comparable performance. The higher negative agreement (91.1%) compared with positive agreement (79.0%) suggests that the assays were more consistent in identifying negative samples than positive samples. This observation is consistent with the pattern of discordant results, where a greater number of samples tested positive on the Bionote FMD NSP Ab ELISA but negative on the PrioCHECK^®^ FMDV NS Ab ELISA.

The Bionote FMD NSP Ab ELISA demonstrated high relative sensitivity (88.7%) and specificity (87.1%) when compared with the PrioCHECK^®^ FMDV NS Ab ELISA, suggesting good ability to identify both positive and negative samples under field conditions.

The Cohen’s kappa value of 0.7 between the Bionote FMD NSP Ab and PrioCHECK^®^ FMDV NS Ab ELISAs indicated substantial agreement between these two assays. Similar kappa-based comparisons between NSP ELISAs have been reported in several studies (Browning et al. 2020; Ruuri et al. 2015; Seeyo et al. 2024), providing useful context for interpreting assay agreement across different epidemiological settings. However, direct comparisons between studies should be interpreted cautiously because agreement estimates are influenced by study design, population structure, disease prevalence, and sample selection strategies. Ruuri et al. (2015), working in Kenya, reported agreement between NSP ELISA assays in samples collected from areas with a high prevalence of FMD, which may increase the proportion of positive samples and influence agreement estimates. Similarly, Seeyo et al. (2024) evaluated NSP ELISA performance using samples from both naturally and experimentally infected animals, which may not fully represent infection dynamics in uncontrolled, real-world conditions. In comparison, our study evaluated field-collected samples under routine diagnostic conditions, which may account for differences in agreement estimates observed across studies. These variations highlight the influence of study design, population structure, and infection dynamics on NSP ELISA performance and underscore the importance of interpreting kappa values within their specific epidemiological context.

Our investigation revealed discrepancies between the two NSP ELISA assays, suggesting possible variability in the performance of these diagnostic tests. The variations in assay design, including the mechanism (blocking vs. competitive), the specific antigens used (3ABC for PrioCHECK FMDV NS Ab ELISA and recombinant 3ABC for Bionote FMD NSP Ab ELISA), and the incubation times, may contribute to the observed variability in results (Bionote 2024; Thermo Fisher Scientific 2024). Similar discrepancies between NSP ELISAs have previously been reported by Ruuri et al. (2015), who reported differences in classification of samples between the Anigen FMD Ab ELISA and the PrioCHECK^®^ FMDV NS Ab ELISA. In serological testing, these discrepancies are not unusual and emphasise the value of validation studies to evaluate test robustness in various epidemiological settings (Clavijo et al. 2004).

The WOAH Terrestrial Manual recommends the concurrent use of two NSP assays to improve confidence in the serological diagnosis of FMD. In this context, the Bionote FMD NSP Ab ELISA may serve as an additional NSP ELISA alongside the routinely used PrioCHECK^®^ FMDV NS Ab ELISA. Where both assays are available, samples may initially be screened using one assay and subsequently tested using the second assay for confirmation. In such a serial testing approach, concordant positive results would provide greater diagnostic confidence than reliance on a single assay alone. Furthermore, the availability of an alternative NSP ELISA would strengthen testing capacity and provide continuity during periods of reagent shortages or supply-chain disruptions affecting either assay.

### Limitations

This study has several limitations. First, not all serum samples were tested against all three SAT serotypes by SPCE. Most samples were tested using the SAT-2 antigen, whereas only a limited number were tested against the SAT-1 and SAT-3 antigens, all of which yielded negative results. Consequently, the available SPCE data were insufficient to comprehensively evaluate NSP ELISA performance across all three SAT serotypes. Second, samples with contamination, moderate to severe haemolysis, or insufficient volume were excluded prior to testing and could not be statistically compared with included samples because no assay results were available for these specimens. Third, infection status was not confirmed using molecular methods, virus isolation, or virus neutralisation testing. Consequently, the true infection status of individual animals could not be established, and the diagnostic sensitivity and specificity of the Bionote FMD NSP Ab ELISA were estimated using the PrioCHECK^®^ FMDV NS Ab ELISA as the reference method rather than an independent biological gold standard. While this approach is appropriate for assessing equivalence between the two NSP ELISAs, it does not permit definitive evaluation of the true diagnostic accuracy of the Bionote FMD NSP Ab ELISA.

## Conclusion

This study represents the first comparison of the Bionote FMD NSP Ab ELISA and the PrioCHECK^®^ FMDV NS Ab ELISA using field sera from cattle exposed to FMDV SAT serotypes in South Africa. The Bionote FMD NSP Ab ELISA demonstrated substantial agreement with the PrioCHECK^®^ FMDV NS Ab ELISA, high relative sensitivity and specificity, and excellent discriminatory performance in ROC analysis. Although differences between the assays were observed, the findings indicate that the Bionote FMD NSP Ab ELISA performs comparably to the PrioCHECK^®^ FMDV NS Ab ELISA and may serve as a useful complementary NSP ELISA for FMD surveillance and diagnostic programmes. Further studies incorporating independent reference methods and additional sample populations would assist in fully characterising its diagnostic performance under diverse epidemiological conditions.

## Supplementary Information

Below is the link to the electronic supplementary material.

Supplementary Material 1

Supplementary Material 2

## Data Availability

All data generated and analysed during this study are available from the corresponding author on reasonable request.
