# Supplementary material for: Comparative evaluation of bionote FMD NSP Ab and PrioCHECK® FMDV NS Ab ELISA kits for detection of foot-and-mouth disease virus non-structural proteins antibodies in SAT serotypes
Source: Vet Res Commun. 2026 Sep 22;50(6):585. doi: 10.1007/s11259-026-11536-2 (PMC13597599; doi:10.1007/s11259-026-11536-2)
Supplement: Supplementary file 1 [file 11259_2026_11536_MOESM1_ESM.docx]

**Supplementary Table S1** Relationship between SAT-2 SPCE results and NSP ELISA outcomes

| SAT-2 SPCE Status | *n* | PrioCHECK positive, *n* (%) | Bionote positive, *n* (%) |
| --- | --- | --- | --- |
| Positive | 57 | 38 (66.7) | 40 (70.2) |
| Inconclusive | 26 | 10 (38.5) | 9 (34.6) |
| Negative | 258 | 25 (9.7) | 48 (18.6) |
