# Supplementary material for: Comparative evaluation of bionote FMD NSP Ab and PrioCHECK® FMDV NS Ab ELISA kits for detection of foot-and-mouth disease virus non-structural proteins antibodies in SAT serotypes
Source: Vet Res Commun. 2026 Sep 22;50(6):585. doi: 10.1007/s11259-026-11536-2 (PMC13597599; doi:10.1007/s11259-026-11536-2)
Supplement: Supplementary file 2 [file 11259_2026_11536_MOESM2_ESM.docx]

**Supplementary Table S2** Raw data for serum samples collected from unvaccinated cattle during routine FMD sero-surveillance in areas with reported SAT-1, SAT-2, and SAT-3 outbreaks. The table presents NSP ELISA results for all 401 serum samples together with the available SAT-2 SPCE results. Grey background shading represents the percentage inhibition values, with darker shading indicating higher values and lighter shading indicating lower values. Red, green, and blue font colours indicate positive, negative, and inconclusive results, respectively.

| **Lab No.** | **PCHECK_PI** | **BNOTE_PI** | **SPCE_PI** |
| --- | --- | --- | --- |
| **TAD 23/21-26** | **95.80** | **94.16** |  |
| **TAD 23/170-30** | **89.82** | **94.56** | **54.00** |
| **TAD 23/0068-25** | **87.05** | **94.22** | **61.00** |
| **TAD 23/21-32** | **86.76** | **92.63** |  |
| **TAD 23/0066-19** | **85.59** | **94.58** | **78.00** |
| **TAD 23/0072-18** | **85.15** | **94.57** | **74.00** |
| **TAD 23/21-17** | **84.77** | **85.18** |  |
| **TAD 23/21-24** | **84.40** | **94.53** |  |
| **TAD 23/170-16** | **83.88** | **94.06** | **79.00** |
| **TAD 23/21-27** | **83.82** | **78.12** |  |
| **TAD 23/21-29** | **83.41** | **75.09** |  |
| **TAD 23/0066-26** | **82.72** | **58.82** | **62.00** |
| **TAD 23/21-35** | **82.60** | **77.68** |  |
| **TAD 23/170-21** | **82.54** | **94.39** | **79.00** |
| **TAD 23/0068-22** | **82.39** | **62.06** | **58.00** |
| **TAD 23/21-21** | **81.63** | **92.10** |  |
| **TAD 23/169-07** | **80.37** | **82.14** | **53.00** |
| **TAD 23/170-19** | **80.18** | **91.12** | **35.00** |
| **TAD 23/0019-01** | **80.17** | **96.09** | **25.00** |
| **TAD 23/0072-08** | **78.77** | **95.13** | **63.00** |
| **TAD 23/21-4** | **78.77** | **67.78** |  |
| **TAD 23/0019-05** | **78.72** | **95.90** | **5.00** |
| **TAD 23/169-21** | **77.74** | **61.37** | **47.00** |
| **TAD 23/21-40** | **77.60** | **81.74** |  |
| **TAD 23/21-16** | **77.48** | **72.95** |  |
| **TAD 23/0068-09** | **76.75** | **92.98** | **63.00** |
| **TAD 23/170-06** | **76.49** | **77.67** | **68.00** |
| **TAD 23/0167-29** | **76.41** | **35.08** | **61.00** |
| **TAD 23/0019-21** | **76.27** | **93.88** | **34.00** |
| **TAD 23/21-39** | **75.53** | **90.26** |  |
| **TAD 23/21-2** | **75.44** | **82.88** |  |
| **TAD 23/21-33** | **75.39** | **80.00** |  |
| **TAD 23/21-34** | **74.95** | **76.55** |  |
| **TAD 23/0072-17** | **74.55** | **92.71** | **61.00** |
| **TAD 23/21-10** | **74.13** | **85.28** |  |
| **TAD 23/21-37** | **73.96** | **66.67** |  |
| **TAD 23/21-31** | **73.49** | **75.28** |  |
| **TAD 23/0068-10** | **72.80** | **95.42** | **64.00** |
| **TAD 23/170-17** | **72.62** | **90.00** | **74.00** |
| **TAD 23/0072-07** | **72.47** | **93.43** | **61.00** |
| **TAD 23/0068-08** | **72.35** | **94.29** | **66.00** |
| **TAD 23/0019-02** | **72.30** | **93.44** | **29.00** |
| **TAD 23/21-22** | **72.23** | **86.93** |  |
| **TAD 23/0068-12** | **72.18** | **94.25** | **75.00** |
| **TAD 23/0002-13** | **71.53** | **76.60** | **22.00** |
| **TAD 23/0068-18** | **71.22** | **77.69** | **60.00** |
| **TAD 23/0002-25** | **71.05** | **60.13** | **-25.00** |
| **TAD 23/0019-07** | **71.00** | **95.08** | **22.00** |
| **TAD 23/0165-22** | **70.73** | **87.63** | **56.00** |
| **TAD 23/0066-23** | **70.72** | **94.64** | **76.00** |
| **TAD 23/0072-03** | **69.55** | **72.89** | **70.00** |
| **TAD 23/169-03** | **68.89** | **72.54** | **48.00** |
| **TAD 23/0072-06** | **68.58** | **85.74** | **78.00** |
| **TAD 23/0068-01** | **68.42** | **74.84** | **65.00** |
| **TAD 23/0167-31** | **67.98** | **89.73** | **67.00** |
| **TAD 23/170-22** | **67.64** | **84.03** | **33.00** |
| **TAD 23/0072-29** | **66.77** | **94.60** | **66.00** |
| **TAD 23/21-38** | **66.45** | **83.81** |  |
| **TAD 23/0066-21** | **66.38** | **92.45** | **35.00** |
| **TAD 23/0167-30** | **66.34** | **84.78** | **62.00** |
| **TAD 23/21-6** | **66.33** | **57.92** |  |
| **TAD 23/0068-29** | **66.04** | **92.49** | **68.00** |
| **TAD 23/0071-12** | **65.81** | **94.54** |  |
| **TAD 23/0167-33** | **65.63** | **89.43** | **69.00** |
| **TAD 23/0066-04** | **65.57** | **94.36** | **50.00** |
| **TAD 23/0072-16** | **65.42** | **93.72** | **38.00** |
| **TAD 23/169-06** | **64.75** | **51.96** | **51.00** |
| **TAD 23/0165-08** | **64.38** | **68.92** | **0.00** |
| **TAD 23/0066-16** | **64.31** | **92.53** | **68.00** |
| **TAD 23/0002-14** | **63.67** | **31.76** | **27.00** |
| **TAD 23/0068-16** | **63.10** | **77.86** | **39.00** |
| **TAD 23/0165-27** | **63.08** | **6.76** | **63.00** |
| **TAD 23/169-29** | **61.94** | **55.94** | **62.00** |
| **TAD 23/170-26** | **61.03** | **93.89** | **62.00** |
| **TAD 23/0165-12** | **60.72** | **44.37** | **52.00** |
| **TAD 23/21-18** | **60.69** | **76.40** |  |
| **TAD 23/169-18** | **60.60** | **47.83** | **58.00** |
| **TAD 23/21-41** | **60.13** | **56.41** |  |
| **TAD 23/21-5** | **59.52** | **50.16** |  |
| **TAD 23/21-30** | **58.45** | **55.09** |  |
| **TAD 23/21-14** | **57.94** | **62.85** |  |
| **TAD 23/21-1** | **57.77** | **43.64** |  |
| **TAD 23/0165-01** | **57.60** | **25.51** | **10.00** |
| **TAD 23/21-23** | **57.43** | **52.86** |  |
| **TAD 23/0066-22** | **57.15** | **82.55** | **65.00** |
| **TAD 23/0068-21** | **57.12** | **84.89** | **6.00** |
| **TAD 23/21-15** | **56.88** | **68.63** |  |
| **TAD 23/169-30** | **56.47** | **35.48** | **47.00** |
| **TAD 23/0066-01** | **56.39** | **96.56** | **61.00** |
| **TAD 23/0019-13** | **56.07** | **61.35** | **5.00** |
| **TAD 23/169-15** | **55.96** | **84.59** | **52.00** |
| **TAD 23/0066-17** | **55.82** | **72.94** | **74.00** |
| **TAD 23/0066-18** | **55.79** | **72.84** | **65.00** |
| **TAD 23/0066-30** | **55.79** | **-1.05** | **75.00** |
| **TAD 23/21-20** | **55.37** | **71.09** |  |
| **TAD 23/0066-25** | **54.95** | **59.18** | **80.00** |
| **TAD 23/0067-06** | **54.30** | **16.80** | **12.00** |
| **TAD 23/0165-14** | **54.20** | **42.50** | **69.00** |
| **TAD 23/170-25** | **54.12** | **56.67** | **46.00** |
| **TAD 23/21-3** | **53.55** | **85.95** |  |
| **TAD 23/0072-11** | **52.90** | **93.59** | **74.00** |
| **TAD 23/0019-14** | **51.84** | **96.06** | **23.00** |
| **TAD 23/0167-22** | **51.62** | **23.68** | **51.00** |
| **TAD 23/0066-06** | **51.37** | **85.31** | **45.00** |
| **TAD 23/21-28** | **50.44** | **52.38** |  |
| **TAD 23/0068-05** | **50.29** | **68.60** | **66.00** |
| **TAD 23/0070-14** | **49.67** | **33.54** | **28.00** |
| **TAD 23/0072-24** | **49.48** | **32.86** | **73.00** |
| **TAD 23/21-7** | **49.08** | **58.90** |  |
| **TAD 23/21-36** | **49.03** | **4.98** |  |
| **TAD 23/0167-28** | **48.76** | **5.23** | **65.00** |
| **TAD 23/0069-16** | **48.29** | **53.39** | **3.00** |
| **TAD 23/0068-30** | **47.87** | **23.40** | **73.00** |
| **TAD 23/0070-18** | **47.84** | **47.84** | **41.00** |
| **TAD 23/0019-30** | **47.37** | **29.10** | **31.00** |
| **TAD 23/0072-09** | **47.37** | **70.93** | **69.00** |
| **TAD 23/0019-20** | **47.10** | **30.55** | **23.00** |
| **TAD 23/0165-13** | **46.59** | **70.07** | **66.00** |
| **TAD 23/0019-16** | **46.43** | **47.41** | **19.00** |
| **TAD 23/0165-15** | **46.31** | **63.66** | **75.00** |
| **TAD 23/0067-26** | **46.17** | **10.10** | **23.00** |
| **TAD 23/170-18** | **45.79** | **54.18** | **72.00** |
| **TAD 23/0066-24** | **45.73** | **63.86** | **79.00** |
| **TAD 23/21-9** | **45.72** | **36.89** |  |
| **TAD 23/170-20** | **45.33** | **49.48** | **62.00** |
| **TAD 23/0070-13** | **45.04** | **22.66** | **5.00** |
| **TAD 23/0002-06** | **45.04** | **19.61** | **7.00** |
| **TAD 23/0002-26** | **44.54** | **16.93** | **-8.00** |
| **TAD 23/170-11** | **44.38** | **39.25** | **26.00** |
| **TAD 23/0067-04** | **44.29** | **27.65** | **-9.00** |
| **TAD 23/0070-07** | **44.19** | **43.05** | **51.00** |
| **TAD 23/21-13** | **43.73** | **36.52** |  |
| **TAD 23/0002-21** | **43.66** | **29.71** | **14.00** |
| **TAD 23/0019-26** | **43.55** | **70.90** | **23.00** |
| **TAD 23/0167-16** | **43.54** | **35.42** | **26.00** |
| **TAD 23/0167-05** | **42.93** | **13.80** | **11.00** |
| **TAD 23/0019-28** | **42.86** | **13.24** | **14.00** |
| **TAD 23/0165-21** | **42.75** | **28.45** | **3.00** |
| **TAD 23/0165-16** | **41.98** | **64.15** | **33.00** |
| **TAD 23/0071-11** | **41.75** | **20.95** |  |
| **TAD 23/170-01** | **41.59** | **-7.17** | **65.00** |
| **TAD 23/0167-09** | **41.52** | **6.27** | **13.00** |
| **TAD 23/0068-17** | **41.40** | **43.85** | **22.00** |
| **TAD 23/21-11** | **41.16** | **45.36** |  |
| **TAD 23/0067-16** | **40.74** | **53.53** | **-2.00** |
| **TAD 23/0066-13** | **40.65** | **73.74** | **-5.00** |
| **TAD 23/21-8** | **40.60** | **51.17** |  |
| **TAD 23/0165-28** | **39.37** | **70.71** | **27.00** |
| **TAD 23/0069-26** | **39.29** | **70.06** | **26.00** |
| **TAD 23/21-12** | **39.14** | **41.38** |  |
| **TAD 23/0165-11** | **38.82** | **33.33** | **29.00** |
| **TAD 23/0067-22** | **38.71** | **25.82** | **-2.00** |
| **TAD 23/0067-12** | **38.57** | **4.22** | **25.00** |
| **TAD 23/0069-22** | **38.19** | **66.95** | **25.00** |
| **TAD 23/0067-10** | **37.69** | **40.23** | **3.00** |
| **TAD 23/0070-28** | **37.59** | **22.72** | **-1.00** |
| **TAD 23/0072-04** | **37.39** | **34.30** | **32.00** |
| **TAD 23/0019-06** | **37.21** | **53.69** | **26.00** |
| **TAD 23/0069-18** | **36.84** | **44.43** | **32.00** |
| **TAD 23/0167-02** | **36.79** | **-7.25** | **14.00** |
| **TAD 23/0070-11** | **36.52** | **49.44** | **44.00** |
| **TAD 23/0068-24** | **36.52** | **46.91** | **37.00** |
| **TAD 23/0069-19** | **36.32** | **20.64** | **31.00** |
| **TAD 23/0069-09** | **36.21** | **50.91** | **41.00** |
| **TAD 23/0067-18** | **35.87** | **18.07** | **23.00** |
| **TAD 23/0002-17** | **35.81** | **52.55** | **-10.00** |
| **TAD 23/0067-24** | **35.58** | **16.86** | **9.00** |
| **TAD 23/0067-25** | **35.47** | **24.05** | **26.00** |
| **TAD 23/0067-17** | **35.47** | **22.42** | **18.00** |
| **TAD 23/0019-11** | **35.16** | **67.21** | **18.00** |
| **TAD 23/0019-10** | **34.99** | **28.28** | **18.00** |
| **TAD 23/0019-25** | **34.95** | **32.76** | **25.00** |
| **TAD 23/169-17** | **34.93** | **17.84** | **37.00** |
| **TAD 23/0002-19** | **34.84** | **33.04** | **3.00** |
| **TAD 23/0068-27** | **34.79** | **77.66** | **47.00** |
| **TAD 23/0070-01** | **34.66** | **27.83** | **38.00** |
| **TAD 23/21-19** | **34.48** | **39.49** |  |
| **TAD 23/0068-04** | **34.47** | **33.25** | **-5.00** |
| **TAD 23/0067-15** | **34.27** | **31.05** | **31.00** |
| **TAD 23/0067-19** | **34.23** | **19.15** | **29.00** |
| **TAD 23/0067-05** | **34.12** | **30.26** | **12.00** |
| **TAD 23/0002-07** | **34.05** | **36.70** | **-6.00** |
| **TAD 23/170-08** | **33.75** | **30.30** | **14.00** |
| **TAD 23/169-20** | **33.68** | **22.97** | **36.00** |
| **TAD 23/169-27** | **33.63** | **2.65** | **59.00** |
| **TAD 23/0069-28** | **33.53** | **22.40** | **8.00** |
| **TAD 23/0068-23** | **33.44** | **48.32** | **39.00** |
| **TAD 23/170-23** | **33.43** | **36.58** | **13.00** |
| **TAD 23/0167-27** | **33.41** | **-3.30** | **15.00** |
| **TAD 23/0072-22** | **33.32** | **25.15** | **16.00** |
| **TAD 23/0002-12** | **33.30** | **7.91** | **19.00** |
| **TAD 23/0068-14** | **33.16** | **43.88** | **54.00** |
| **TAD 23/0070-30** | **33.10** | **36.15** | **26.00** |
| **TAD 23/0071-24** | **33.10** | **15.76** |  |
| **TAD 23/0069-25** | **33.03** | **47.68** | **35.00** |
| **TAD 23/0167-06** | **33.01** | **41.12** | **12.00** |
| **TAD 23/0067-09** | **32.97** | **17.09** | **21.00** |
| **TAD 23/169-05** | **32.83** | **36.03** | **45.00** |
| **TAD 23/0067-28** | **32.39** | **25.72** | **33.00** |
| **TAD 23/0165-03** | **32.34** | **25.47** | **1.00** |
| **TAD 23/0070-02** | **32.05** | **37.49** | **22.00** |
| **TAD 23/0019-15** | **31.94** | **34.11** | **30.00** |
| **TAD 23/0068-19** | **31.74** | **49.94** | **54.00** |
| **TAD 23/0165-18** | **31.71** | **19.61** | **27.00** |
| **TAD 23/0019-03** | **31.38** | **50.60** | **22.00** |
| **TAD 23/0018-07** | **31.38** | **45.98** | **18.00** |
| **TAD 23/0070-09** | **31.18** | **21.98** | **7.00** |
| **TAD 23/0067-01** | **31.16** | **91.67** | **49.00** |
| **TAD 23/0019-12** | **31.05** | **20.18** | **8.00** |
| **TAD 23/21-25** | **31.03** | **81.83** |  |
| **TAD 23/0019-08** | **30.81** | **36.66** | **24.00** |
| **TAD 23/0002-29** | **30.73** | **19.97** | **2.00** |
| **TAD 23/0002-09** | **30.69** | **11.90** | **27.00** |
| **TAD 23/0069-15** | **30.67** | **31.56** | **-4.00** |
| **TAD 23/0067-30** | **30.66** | **11.18** | **37.00** |
| **TAD 23/0019-29** | **30.54** | **27.87** | **29.00** |
| **TAD 23/0002-01** | **30.48** | **34.31** | **-24.00** |
| **TAD 23/0018-08** | **30.42** | **36.63** | **29.00** |
| **TAD 23/0066-11** | **30.33** | **92.75** | **36.00** |
| **TAD 23/0019-18** | **30.28** | **25.13** | **24.00** |
| **TAD 23/0165-29** | **30.28** | **26.47** | **3.00** |
| **TAD 23/0019-27** | **30.10** | **70.08** | **36.00** |
| **TAD 23/0068-11** | **29.96** | **25.29** | **27.00** |
| **TAD 23/0072-26** | **29.73** | **12.79** | **27.00** |
| **TAD 23/0019-22** | **29.69** | **18.79** | **27.00** |
| **TAD 23/169-10** | **29.69** | **19.21** | **53.00** |
| **TAD 23/169-11** | **29.69** | **25.20** | **17.00** |
| **TAD 23/0070-24** | **29.53** | **32.45** | **54.00** |
| **TAD 23/0071-29** | **29.40** | **49.35** |  |
| **TAD 23/0071-20** | **29.22** | **18.54** |  |
| **TAD 23/0002-05** | **29.04** | **30.03** | **21.00** |
| **TAD 23/0066-12** | **28.81** | **43.66** | **80.00** |
| **TAD 23/0067-23** | **28.59** | **26.18** | **-4.00** |
| **TAD 23/0066-27** | **28.58** | **38.37** | **47.00** |
| **TAD 23/0019-23** | **28.57** | **39.97** | **30.00** |
| **TAD 23/0071-17** | **28.27** | **21.63** |  |
| **TAD 23/0018-09** | **28.21** | **29.48** | **16.00** |
| **TAD 23/0069-27** | **27.96** | **15.59** | **34.00** |
| **TAD 23/0071-23** | **27.90** | **36.79** |  |
| **TAD 23/169-16** | **27.73** | **46.49** | **64.00** |
| **TAD 23/0070-26** | **27.64** | **42.99** | **49.00** |
| **TAD 23/169-28** | **27.64** | **10.80** | **1.00** |
| **TAD 23/0070-16** | **27.51** | **39.78** | **38.00** |
| **TAD 23/0069-08** | **27.49** | **27.39** | **44.00** |
| **TAD 23/0019-04** | **27.39** | **33.10** | **30.00** |
| **TAD 23/0068-03** | **27.29** | **10.45** | **5.00** |
| **TAD 23/0071-19** | **27.27** | **16.23** |  |
| **TAD 23/0167-13** | **26.99** | **2.10** | **15.00** |
| **TAD 23/0067-13** | **26.99** | **44.80** | **23.00** |
| **TAD 23/0072-21** | **26.96** | **5.17** | **18.00** |
| **TAD 23/0019-19** | **26.95** | **10.94** | **20.00** |
| **TAD 23/0072-02** | **26.94** | **-9.32** | **21.00** |
| **TAD 23/0165-07** | **26.83** | **34.40** | **-12.00** |
| **TAD 23/0069-17** | **26.80** | **36.69** | **35.00** |
| **TAD 23/0019-24** | **26.76** | **14.66** | **35.00** |
| **TAD 23/0069-20** | **26.75** | **14.48** | **26.00** |
| **TAD 23/0070-23** | **26.68** | **51.69** | **59.00** |
| **TAD 23/0067-03** | **26.37** | **16.70** | **37.00** |
| **TAD 23/0072-14** | **26.36** | **24.69** | **39.00** |
| **TAD 23/0068-13** | **26.27** | **18.51** | **82.00** |
| **TAD 23/0167-21** | **26.21** | **15.03** | **11.00** |
| **TAD 23/0070-21** | **26.00** | **31.62** | **51.00** |
| **TAD 23/0070-08** | **25.86** | **51.37** | **23.00** |
| **TAD 23/0165-05** | **25.86** | **25.21** | **31.00** |
| **TAD 23/0072-10** | **25.75** | **42.02** | **31.00** |
| **TAD 23/0070-29** | **25.65** | **29.01** | **-2.00** |
| **TAD 23/0066-14** | **25.54** | **50.79** | **21.00** |
| **TAD 23/0067-29** | **25.54** | **20.95** | **15.00** |
| **TAD 23/170-13** | **25.51** | **26.79** | **54.00** |
| **TAD 23/0069-02** | **25.43** | **28.70** | **13.00** |
| **TAD 23/0066-09** | **25.20** | **42.18** | **61.00** |
| **TAD 23/0167-12** | **25.03** | **4.82** | **23.00** |
| **TAD 23/0002-22** | **25.02** | **8.20** | **24.00** |
| **TAD 23/0018-12** | **24.97** | **36.99** | **15.00** |
| **TAD 23/0018-14** | **24.88** | **30.59** | **23.00** |
| **TAD 23/0165-04** | **24.84** | **30.67** | **15.00** |
| **TAD 23/0067-14** | **24.75** | **37.39** | **14.00** |
| **TAD 23/0066-05** | **24.71** | **29.10** | **13.00** |
| **TAD 23/0068-15** | **24.22** | **35.14** | **41.00** |
| **TAD 23/0167-20** | **24.09** | **38.04** | **14.00** |
| **TAD 23/0070-15** | **23.97** | **37.46** | **-5.00** |
| **TAD 23/0167-24** | **23.54** | **70.71** | **26.00** |
| **TAD 23/0002-18** | **23.53** | **12.39** | **14.00** |
| **TAD 23/0071-14** | **23.32** | **19.41** |  |
| **TAD 23/0167-14** | **23.23** | **27.18** | **20.00** |
| **TAD 23/0071-25** | **23.06** | **23.06** |  |
| **TAD 23/170-03** | **23.00** | **-0.17** | **-8.00** |
| **TAD 23/0068-07** | **22.93** | **25.67** | **16.00** |
| **TAD 23/0018-06** | **22.88** | **28.46** | **20.00** |
| **TAD 23/0072-05** | **22.83** | **35.06** | **68.00** |
| **TAD 23/0071-21** | **22.81** | **31.00** |  |
| **TAD 23/0165-24** | **22.68** | **70.71** | **12.00** |
| **TAD 23/0066-15** | **22.56** | **28.28** | **64.00** |
| **TAD 23/0167-07** | **22.53** | **41.45** | **34.00** |
| **TAD 23/0070-03** | **22.44** | **10.03** | **7.00** |
| **TAD 23/170-07** | **22.31** | **-16.03** | **20.00** |
| **TAD 23/0067-07** | **22.27** | **88.46** | **-6.00** |
| **TAD 23/0067-08** | **22.12** | **-5.46** | **70.00** |
| **TAD 23/0068-02** | **22.10** | **16.96** | **22.00** |
| **TAD 23/0072-13** | **22.08** | **46.83** | **37.00** |
| **TAD 23/169-09** | **22.01** | **20.52** | **6.00** |
| **TAD 23/0167-03** | **21.99** | **47.42** | **2.00** |
| **TAD 23/0167-11** | **21.89** | **7.06** | **7.00** |
| **TAD 23/0070-17** | **21.86** | **21.27** | **48.00** |
| **TAD 23/0002-04** | **21.41** | **39.80** | **21.00** |
| **TAD 23/0072-30** | **21.34** | **69.46** | **12.00** |
| **TAD 23/0167-18** | **20.98** | **-2.85** | **16.00** |
| **TAD 23/169-08** | **20.88** | **49.95** | **54.00** |
| **TAD 23/0165-20** | **20.75** | **13.20** | **31.00** |
| **TAD 23/0068-26** | **20.71** | **31.21** | **52.00** |
| **TAD 23/0072-19** | **20.60** | **5.72** | **55.00** |
| **TAD 23/0018-04** | **20.49** | **46.67** | **3.00** |
| **TAD 23/169-19** | **20.46** | **7.53** | **29.00** |
| **TAD 23/0165-25** | **20.35** | **26.47** | **30.00** |
| **TAD 23/0069-03** | **20.16** | **41.51** | **41.00** |
| **TAD 23/0072-20** | **20.03** | **85.25** | **53.00** |
| **TAD 23/169-24** | **19.82** | **-1.25** | **-1.00** |
| **TAD 23/0071-15** | **19.78** | **12.12** |  |
| **TAD 23/0018-13** | **19.76** | **31.08** | **21.00** |
| **TAD 23/0071-26** | **19.64** | **11.77** |  |
| **TAD 23/0018-20** | **19.50** | **42.52** | **5.00** |
| **TAD 23/0018-17** | **19.41** | **47.09** | **3.00** |
| **TAD 23/170-09** | **19.28** | **-17.37** | **1.00** |
| **TAD 23/169-13** | **19.26** | **24.46** | **53.00** |
| **TAD 23/0167-32** | **19.19** | **29.56** | **-3.00** |
| **TAD 23/0071-22** | **19.06** | **23.02** |  |
| **TAD 23/0072-01** | **18.90** | **10.37** | **34.00** |
| **TAD 23/0018-02** | **18.70** | **51.34** | **6.00** |
| **TAD 23/169-26** | **18.69** | **-13.46** | **5.00** |
| **TAD 23/0066-20** | **18.54** | **25.85** | **25.00** |
| **TAD 23/0165-10** | **18.51** | **21.81** | **18.00** |
| **TAD 23/169-23** | **18.45** | **-4.23** | **14.00** |
| **TAD 23/0002-15** | **18.34** | **5.56** | **20.00** |
| **TAD 23/0167-10** | **18.21** | **27.25** | **5.00** |
| **TAD 23/0002-27** | **18.10** | **16.27** | **-8.00** |
| **TAD 23/0072-25** | **18.08** | **19.85** | **20.00** |
| **TAD 23/0069-12** | **18.05** | **12.93** | **31.00** |
| **TAD 23/0069-24** | **18.02** | **6.27** | **38.00** |
| **TAD 23/0018-11** | **17.99** | **26.57** | **16.00** |
| **TAD 23/0071-08** | **17.95** | **12.47** |  |
| **TAD 23/0019-09** | **17.62** | **21.41** | **23.00** |
| **TAD 23/0165-06** | **17.23** | **37.04** | **14.00** |
| **TAD 23/0070-10** | **17.04** | **40.74** | **16.00** |
| **TAD 23/0165-23** | **17.02** | **38.34** | **11.00** |
| **TAD 23/0002-10** | **16.94** | **13.24** | **9.00** |
| **TAD 23/0002-16** | **16.70** | **27.88** | **5.00** |
| **TAD 23/0070-05** | **16.47** | **40.87** | **2.00** |
| **TAD 23/0002-24** | **16.43** | **24.97** | **21.00** |
| **TAD 23/0069-29** | **16.32** | **53.30** | **-3.00** |
| **TAD 23/0072-28** | **16.30** | **6.90** | **21.00** |
| **TAD 23/0069-07** | **16.30** | **24.60** | **35.00** |
| **TAD 23/0067-02** | **16.04** | **12.68** | **42.00** |
| **TAD 23/0071-27** | **15.98** | **24.69** |  |
| **TAD 23/0167-04** | **15.96** | **-21.42** | **25.00** |
| **TAD 23/0069-30** | **15.88** | **7.24** | **11.00** |
| **TAD 23/0072-12** | **15.86** | **16.25** | **23.00** |
| **TAD 23/0167-15** | **15.66** | **16.71** | **8.00** |
| **TAD 23/169-02** | **15.51** | **21.26** | **-18.00** |
| **TAD 23/170-15** | **15.43** | **12.00** | **16.00** |
| **TAD 23/0002-11** | **15.32** | **26.14** | **31.00** |
| **TAD 23/0167-17** | **15.25** | **39.89** | **15.00** |
| **TAD 23/0165-09** | **15.22** | **26.88** | **22.00** |
| **TAD 23/0069-04** | **15.01** | **12.90** | **15.00** |
| **TAD 23/0069-23** | **14.95** | **24.36** | **37.00** |
| **TAD 23/0018-05** | **14.84** | **26.99** | **23.00** |
| **TAD 23/169-01** | **14.80** | **24.65** | **7.00** |
| **TAD 23/169-14** | **14.63** | **69.97** | **2.00** |
| **TAD 23/0018-10** | **14.59** | **22.42** | **12.00** |
| **TAD 23/169-12** | **14.19** | **-79.71** | **0.00** |
| **TAD 23/0069-11** | **14.13** | **14.62** | **15.00** |
| **TAD 23/0070-25** | **14.08** | **42.99** | **54.00** |
| **TAD 23/0165-02** | **13.83** | **8.31** | **8.00** |
| **TAD 23/0071-28** | **13.72** | **-0.03** |  |
| **TAD 23/0018-19** | **13.67** | **34.05** | **16.00** |
| **TAD 23/0067-27** | **13.60** | **23.76** | **3.00** |
| **TAD 23/0069-13** | **13.33** | **24.78** | **6.00** |
| **TAD 23/0067-21** | **13.30** | **43.89** | **38.00** |
| **TAD 23/0167-25** | **13.26** | **26.40** | **18.00** |
| **TAD 23/0167-01** | **12.42** | **34.78** | **11.00** |
| **TAD 23/0002-23** | **12.07** | **14.22** | **17.00** |
| **TAD 23/0167-19** | **11.84** | **19.09** | **59.00** |
| **TAD 23/0167-08** | **11.69** | **2.10** | **8.00** |
| **TAD 23/0002-08** | **11.24** | **-2.58** | **-14.00** |
| **TAD 23/0071-05** | **10.71** | **5.37** |  |
| **TAD 23/0165-26** | **10.43** | **16.97** | **9.00** |
| **TAD 23/170-12** | **9.37** | **41.88** | **39.00** |
| **TAD 23/0070-27** | **9.25** | **16.97** | **46.00** |
| **TAD 23/0071-16** | **8.81** | **13.98** |  |
| **TAD 23/0066-02** | **8.71** | **44.29** | **70.00** |
| **TAD 23/0069-01** | **8.40** | **30.22** | **32.00** |
| **TAD 23/0066-03** | **8.08** | **15.67** | **41.00** |
| **TAD 23/0018-16** | **6.66** | **41.73** | **18.00** |
| **TAD 23/0165-17** | **6.29** | **12.24** | **7.00** |
| **TAD 23/0067-20** | **3.67** | **9.15** | **41.00** |
| **TAD 23/0070-19** | **3.21** | **16.10** | **44.00** |
| **TAD 23/0066-07** | **0.58** | **13.75** | **40.00** |
| **TAD 23/0066-28** | **0.05** | **-1.05** | **-13.00** |
| **TAD 23/0070-22** | **0.00** | **50.66** | **40.00** |
| **TAD 23/170-28** | **0.00** | **77.48** | **68.00** |
| **TAD 23/0018-01** | **-11.28** | **11.60** | **16.00** |
| **TAD 23/0018-18** | **-16.38** | **38.40** | **10.00** |
